# Feasibility Study of a Fully Synchronous Virtual Critical Care Elective Focused on Learner Engagement

**DOI:** 10.7759/cureus.25427

**Published:** 2022-05-28

**Authors:** Soyun Michelle Hwang, Ambrose Rice, Serkan Toy, Rachel Levine, Lee Goeddel

**Affiliations:** 1 Anesthesiology and Critical Care, Johns Hopkins University School of Medicine, Baltimore, USA; 2 Anesthesiology, Johns Hopkins University School of Medicine, Baltimore, USA; 3 Internal Medicine, Johns Hopkins University School of Medicine, Baltimore, USA

**Keywords:** innovation in medical education, medical education, learner engagement, virtual learning, critical care, telemedicine

## Abstract

Background: The COVID-19 pandemic disrupted clinical education for medical students. With the rise of variants, meaningful in-person clinical experiences remain threatened. This report describes the design, implementation, and evaluation of a fully synchronous virtual critical care elective for medical students focused on learner engagement.

Methods: The two-week elective was offered during June and July 2020 in the COVID-19 extracorporeal membrane oxygenation (ECMO) unit. Medical students remotely participated in multidisciplinary rounds with the attending physician connected from the bedside via a head-mounted camera providing the first-person video view. Other team members connected outside the negative pressure area. Learners electronically completed daily intensive care unit (ICU) goals sheet (GS) for each patient. The daily completion percentage of the GS assessed the learner engagement, and the learners evaluated the experience with a five-point Likert scale survey.

Results: Nine medical students participated in two separate cohorts. Cohort A had 53 patient encounters, and Cohort B had 45 patient encounters totaling 301.5 total hours of supervised virtual patient interaction. The mean completion percentage of the daily ICU GS for the combined cohorts was 77.8%, (with a standard deviation of 9.6%), with sustained or increased completion from start to finish for all learners. All medical students agreed that the daily ICU GS was helpful for following rounds, organizing patient assessments and plans, and participating in patient care. The majority (88.9%) agreed that the elective increased their comfort in caring for critically ill patients.

Conclusions: During the COVID-19 pandemic, a fully synchronous virtual critical care elective successfully utilized the first-person view and daily ICU GS to promote and assess learner engagement.

## Introduction

COVID-19 restrictions on medical training have prompted the need for innovative approaches to virtual learning. By late March of 2020, nonclinical education transitioned to virtual platforms [1-3]. However, in-person clinical learning opportunities were significantly reduced due to major barriers restricting meaningful transition to a virtual educational experience [4]. The lack of clinical experience has had major consequences on third- and fourth-year medical students including limiting the development of physical exam, technical, and nontechnical skills necessary for clinical competence while also negatively affecting the learners’ mental health [5-7].

COVID-19 variants now increasingly threaten in-person learning opportunities [8]. Critical care experience for medical students is particularly vital [9-11]. A high-quality virtual critical care elective could both address the lack of clinical exposure during COVID-19 restrictions and offer learning opportunities previously inaccessible to many learners. Achieving meaningful virtual learner participation and engagement, however, remains a major challenge to successful virtual clinical electives [12].

During the summer of 2020 amidst the COVID-19 pandemic, we developed a fully synchronous virtual critical care elective for medical students, which focused on optimizing learner engagement. We employed a novel approach to promote and simultaneously assess virtual learner engagement using a daily intensive care unit (ICU) goals sheet (GS), a well-established and easily adaptable tool for improving provider engagement and performance, interdisciplinary communication, and patient outcomes in both ICU and non-ICU settings [13-15].

We hypothesized that completing the daily ICU GS would provide the learner with a structure for building their assessment and plan for critically ill patients and enable them to actively follow the synchronous learning activity. Additionally, the completion percentage could serve as a novel measure of learner engagement by assessing learner conduct, effort, and participation. This report describes the design, implementation, and evaluation of a fully synchronous virtual critical care elective for medical students with a particular focus on learner participation and engagement outcomes.

## Materials and methods

The Johns Hopkins University School of Medicine Institutional Review Board (IRB) approved this study, and the requirement for written informed consent was waived by the IRB.

Course goal and learning objectives

The course goal was to improve the medical students’ preparedness to evaluate critically ill patients through a technically feasible virtual clinical ICU elective. Elective students were expected to achieve the following learning objectives: (1) evaluate critically ill patients during rounds as a part of an interdisciplinary team, (2) devise an appropriate clinical assessment and plan for a critically ill patient, and (3) feel more prepared in caring for critically ill patients.

Course design and implementation

**Figure 1:**
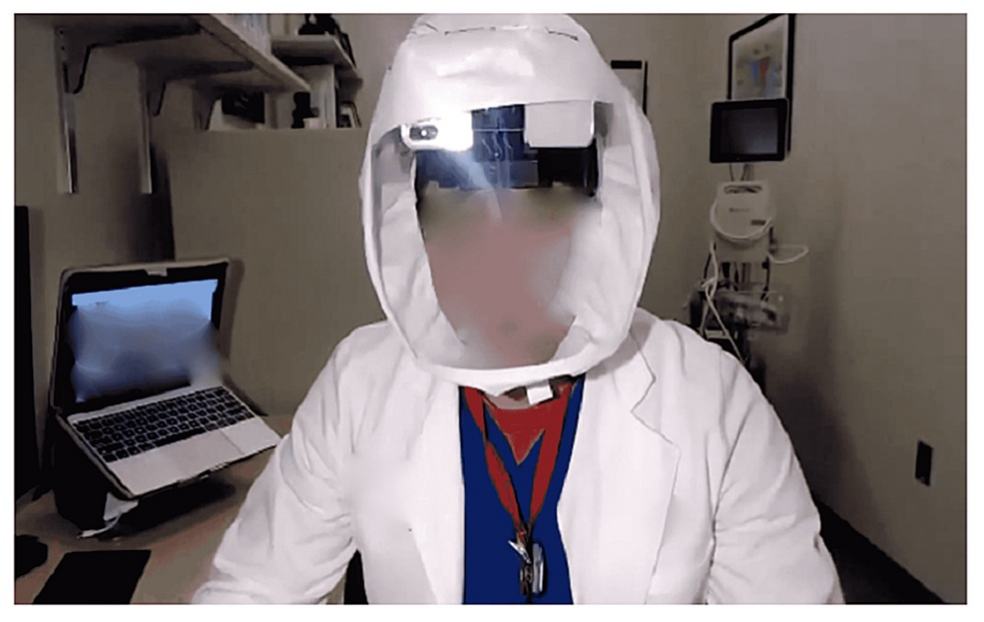
Simulated demonstration of camera phone mounted under personal protective equipment The camera phone on the head mount under the PAPR forehead strap was used for the entirety of the time teaching and rounding in the negative pressure environment. PAPR: Powered air-purifying respirator.

The two-week virtual critical care elective was developed and implemented between June and July 2020 (at which time social distancing and quarantine guidelines were active) at the Johns Hopkins Hospital by the Anesthesiology and Critical Care Medicine (ACCM) Department in the COVID-19 extracorporeal membrane oxygenation (ECMO) ICU at Johns Hopkins Hospital. All virtual technology platforms utilized during the elective were compliant with Health Information Portability and Accountability Act (HIPAA) guidelines. For a full description of the technology and workflow used to establish virtual rounds, see Figure 1 and the appendix for a “how-to” recreate the technologic framework, and to access the daily goal sheets to establish a virtual elective, please refer to the data supplement.

The elective was made available to all third- and fourth-year medical students of Johns Hopkins School of Medicine who were interested in critical care but had no previous ICU experience. Students were geographically located throughout the country. Each student cohort was kept small, given the high acuity of patient care, to allow for optimal virtual interaction with the attending physician and the interdisciplinary ICU team.

Instructional methods

Multidisciplinary virtual bedside teaching rounds

**Figure 2:**
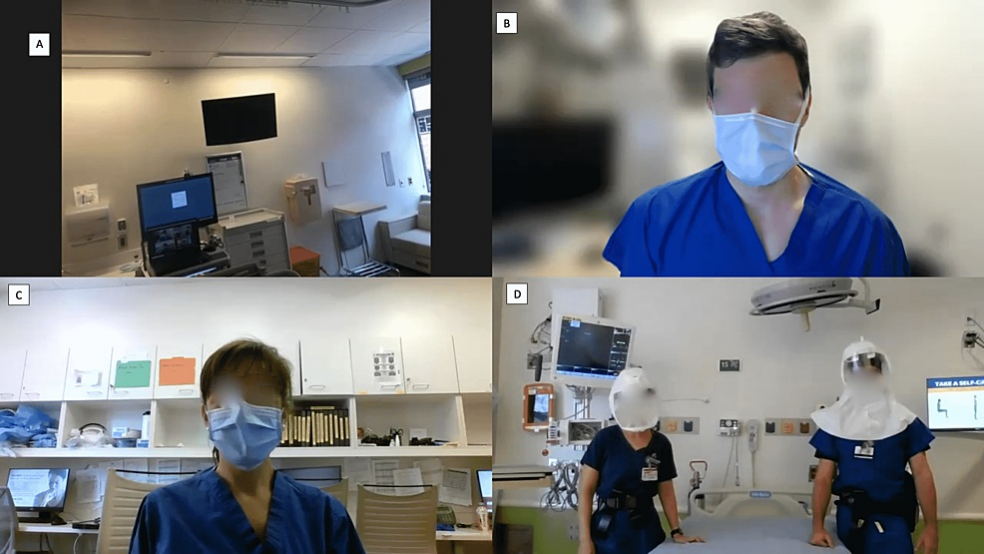
Simulated demonstration of camera phone mounted under PAPR and virtual synchronous bedside rounds Panel (A): A first-person video view from a head-mounted camera phone. Panels (B) and (C): Remote team members and learners connected over Zoom. Panel (D): A view from an additional camera at the bedside mounted from the workstation to provide additional perspective showing the bedside nurse (left) and the bedside attending physician (right) with a head-mounted camera. PAPR: Powered air-purifying respirator.

Each elective day began with morning rounds. All participating medical students joined rounds via the Zoom platform. Resident physicians and physician assistants who were on site in the ICU were socially distanced in a separate room within the ICU and virtually logged in via the Johns Hopkins Institutional Zoom platform. Pharmacists and nutritionists also joined by Zoom remotely. Using a head-mounted first-person view camera under personal protective equipment (PPE), the attending physician provided real-time visual and audio access of the patient from the bedside. The attending physician projected real-time clinical data from medical equipment such as ventilators, medicine pumps, and ECMO machines as well as appropriate physical exam findings. Figure 2 demonstrates a visual example of virtual rounding.

The patient assessment and plan were discussed virtually at the bedside with real-time access to the patient and key clinical data. Nursing and respiratory therapy staff joined rounds in-person at the bedside. Each medical student assessed four to eight patients per day.

Daily ICU Goals Sheet

The daily ICU GS (supplement) was developed by Pronovost et al. at Johns Hopkins Hospital and has shown to improve multiple patient care outcomes [13,14]. Given its established adaptable use, blank daily ICU GSs were delivered to learners to complete for each patient via an electronic database. The database was housed in a HIPAA-protected file-sharing program, and an electronic folder was created for each learner.

Post-virtual Round Debrief Session

After morning multidisciplinary bedside rounds, the medical students virtually participated in an interactive debrief session with the attending physician. During this time, each medical student synthesized a system-based assessment for each patient. The attending physician provided feedback to students and additional clinical teaching. Every component of the virtual critical care elective occurred synchronously in real time with an intentional design to maximize the interaction and learner engagement.

Student assessment and elective grading

During the pandemic, grading throughout the medical school curricula transitioned to pass/fail. To pass the course, students were expected to attend the virtual rounds, engage in the debrief sessions, and attempt to work through the daily ICU GS. Students were instructed that the daily ICU GS was provided as a template to assist them in their learning. If they chose to use it, there was no required level of percent completion.

Elective evaluation methods

The feasibility of the virtual critical care elective was evaluated via a post-elective survey on a five-point Likert scale (“strongly disagree” to “somewhat disagree” to “neutral” to “somewhat agree” to “strongly agree”). The survey questions assessed characteristics of the virtual platform, such as sound quality, video quality, connection quality, ease of following rounds, and educational benefit of the first-person view camera. The survey also included a free-text comment section for medical students to provide anonymous qualitative feedback.

Learner engagement was first evaluated via the completion percentage of the daily ICU GS. The completion percentage was calculated, and its trend was analyzed. Percent completion for all the patients evaluated by each medical student was averaged per day. The daily average completion rate was recorded, compared, and trended throughout the duration of the elective for each medical student. Statistically significant changes in the daily average completion percentage throughout the duration of the elective were analyzed for statistical significance using the student t-test. Learner engagement was also assessed with a post-elective survey on a five-point Likert scale (“strongly disagree” to “somewhat disagree” to “neutral” to “somewhat agree” to “strongly agree”).

## Results

Student characteristics

For the first iteration (Cohort A) of the virtual critical care elective, four medical students (Students A, B, C, and D) participated and completed the elective in a duration of eight days except for Student C who had an excused absence on Day 4 of the elective. For the second iteration (Cohort B) of the virtual critical care elective, five medical students (Students V, W, X, Y, and Z) participated and completed the elective in a duration of seven days, except for Student Z who had an excused absence on Day 7. In total, nine medical students completed the virtual critical care elective.

Among these nine medical students, seven were identified as males and two were females. Five were third-year medical students, and four were fourth-year medical students. The average age was 29.3 years. Seven students have planned to apply for internal medicine (or a related field through internal medicine residency), one decided to apply for anesthesiology, and one has not decided.

Course feasibility

**Figure 3:**
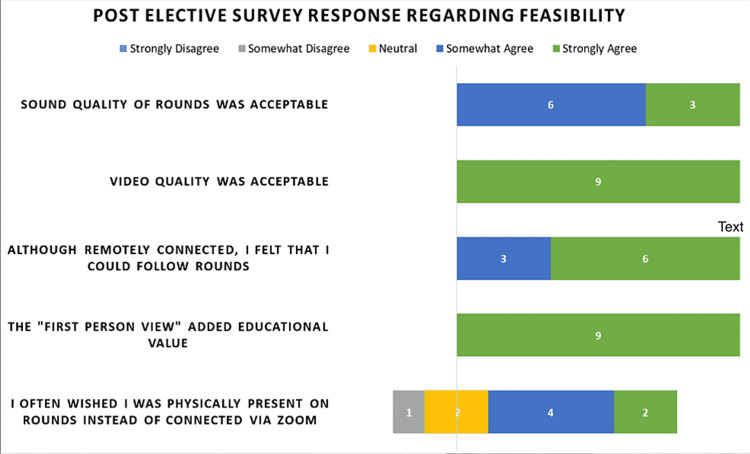
Post-elective survey assessment of the feasibility of the fully synchronous virtual critical care elective virtual platform Results from this survey demonstrate strong feasibility metrics with sound, video quality, and connection to the rounding experience.

The medical students were asked to evaluate the feasibility of the elective upon completion (Figure 3). All nine medical students completed the post-elective survey. All medical students agreed that the sound and video quality of the elective was acceptable and that they could follow rounds. All medical students strongly agreed that the first-person video view added educational value. When asked if they wished to be physically present on rounds instead of being connected virtually, there was a wider range of survey responses: one student somewhat disagreed, two students felt neutral, four students somewhat agreed, and two students strongly agreed.

Patient interactions

Each student in Cohort A had a total of 53 patient encounters during the eight-day elective period (an average of 6.6 patient encounters per day). Each student in Cohort B had a total of 45 patient encounters during the seven-day elective period (an average of 6.4 patient encounters per day). Counting time during rounds and bedside clinical teaching rounds yielded 36 total hours of virtual patient interaction per student in Cohort A and 31.5 hours per student in Cohort B. In sum, 301.5 hours of supervised virtual patient interaction for nine medical students were achieved during the elective offerings.

Learner engagement

**Figure 4:**
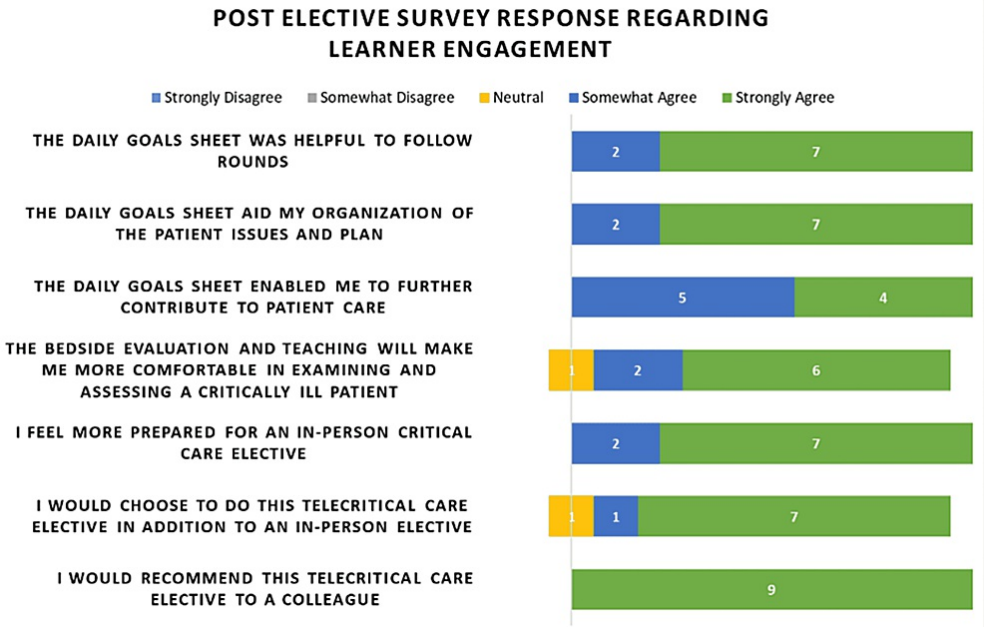
Post-elective survey assessment of the learner engagement of the fully synchronous virtual critical care elective virtual platform Learner engagement is a major challenge in virtual learning. The survey results were all suggestive that the daily goal sheets and organization of this elective successfully engaged the virtual learners.

Learner engagement was first assessed by survey (Figure 4). All medical students agreed that the daily ICU GS was helpful as a template for following rounds and for organizing the patient assessments and plans. All medical students also agreed that the daily ICU GS enabled them to contribute to patient care (n = 4: strongly agreed, n = 5: somewhat agreed). When asked if the bedside evaluation and teaching would make them feel more comfortable in caring for a critically ill patient, six students strongly agreed, two students somewhat agreed, and one student was neutral. All medical students agreed that they felt more prepared for an in-person critical care elective after the virtual critical care elective (n = 7: strongly agreed, and n = 2: somewhat agreed). When asked if they would choose to do this virtual critical care elective in addition to an in-person critical care elective, seven students strongly agreed, one student somewhat agreed, and one student was neutral. All nine medical students strongly agreed that they would recommend the virtual critical care elective to a colleague.

The daily average completion percentages of the daily ICU GS were also analyzed as a measurement of learner participation and engagement. Each medical student had four to eight patient encounters per day. Figure 3 summarizes the daily ICU GS completion percentages per day for each student and demonstrates the trend of completion for each student throughout the elective period. Of note, in Cohort B, Student V’s daily ICU GS data were not included due to technical errors in the collection process. Therefore, only eight data sets were analyzed.

**Figure 5:**
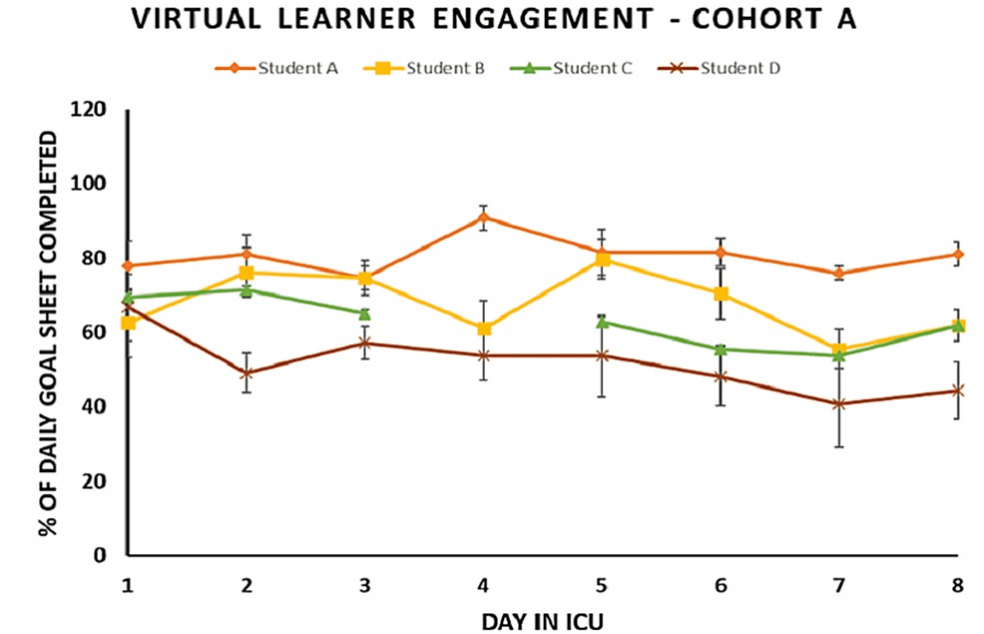
Cohort A ICU daily goals sheet completion percentages also show learner engagement In Cohort A (n = 4), all students showed a sustained level of ICU daily goals sheet completion throughout the duration of the elective when compared to Day 1. This is a metric of sustained learner engagement across the elective.

**Figure 6:**
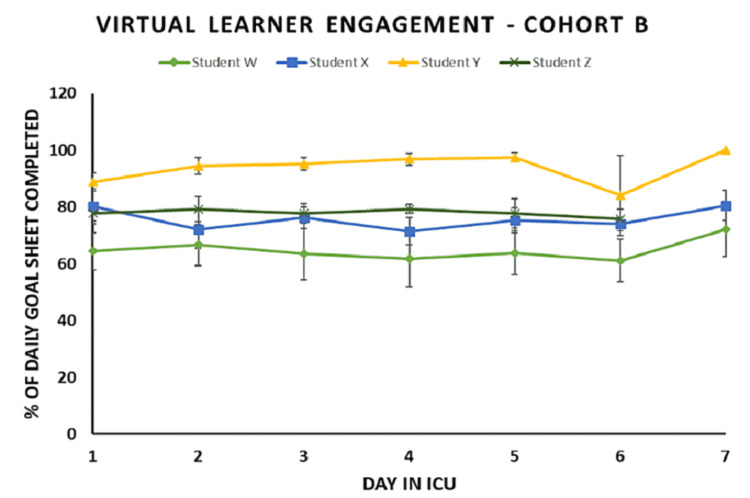
Cohort B ICU daily goals sheet completion percentages as a reflection of learner engagement In Cohort B (n = 4), Student Y showed a significant increase (p < 0.05* and p <0.01**) in daily goals sheet completion on Day 2 and Day 7 compared to Day 1. Student W and Student X also showed a near significant increase in daily goals sheet completion on Day 2 and Day 4, respectively (p = 0.06 and p = 0.07). The sustained goal sheet completion percentages demonstrate sustained learner engagement in Cohort B also.

Daily ICU GS data (Figures 5, 6) show sustained or increased percentage of completion by each student throughout the duration of the elective in both cohorts. The mean completion percentage per day per student ranged from the lowest of 51% to the highest of 93%. The mean completion percentage for the entire elective duration for all the medical students (n = 8) was 78%. In Cohort A (Figure 5), three out of four students demonstrated a sustained level of the daily ICU GS completion throughout the elective when compared to the initial day (Day 1) as well as all preceding days. Student D displayed sustained completion with an average of 53%, and a minimal variation over the course of the elective from Day 1 (66%) was found not to be statistically different from Day 8 (44%) (p = 0.07). In Cohort B (Figure 6), Student Y showed a significant increase in the daily ICU GS completion on Day 2 (p < 0.01) and Day 7 (p = 0.005) compared to Day 1. All the other students in Cohort B demonstrated a sustained level of the daily ICU GS completion.

Anonymous qualitative feedback collected via a post-elective survey provided further insights into the feasibility and learner engagement. Six out of nine students authored feedback. The comments were minimally edited for readability and presented in Table 1.

**Table 1:** Anonymous qualitative learner feedback

S. No	Comments
1	Great first experience in the ICU.
2	A great introduction to rounding as this was my first in-hospital clinical experience.
3	I felt integrated into rounds and we were offered many opportunities to participate.
4	I found the teaching by the residents and the patients’ stories amazing.
5	I loved zooming in with a nurse or a respiratory therapist.
6	The team felt more cohesive because of the intentional reflection, sharing, and learning (during the post-round virtual debrief session).
7	It was great to follow along with some of the heavy conversations with patients and families because those types of conversations are important to learn from examples.
8	While there were some tech issues related to audio/video, they were overall minor and not inherently different from the challenges of any remote conferencing.
9	I almost wished there were more I could do, such as calling family, communicating with consultants, writing notes, etc.
10	The ICU daily goals sheet was helpful to track along (with rounds) but I wasn’t always able to fill in every checkbox.
11	Sometimes the ICU daily goals sheet did not have options that fit every patient, e.g., different types of anticoagulation, antifungals, and adjustments for other medications.

## Discussion

This virtual critical care elective provides several novel contributions. First, this report describes the largest virtual elective in an ICU setting to date that we are aware of, involving nine medical students. Ho et al. recently reported on the first tele ICU pilot rotation involving five medical students, which entailed a four-week course totaling 60 hours of virtual clinical experience per student [16]. While our elective was shorter in overall duration, we provided 36 hours of virtual clinical experience per student on average, which is slightly more averaged per week. Aside from this study, there is a current lack of a new virtual platform in the ICU specifically designed for medical students' education. This is particularly important since the Association of American Medical Colleges (AAMC) published a set of telehealth competencies in the core learning curriculum for medical students in March 2021 [17].

However, we can learn from the rapidly growing practice of virtual ICU platforms across the country [18,19]. Overall, the current practice of tele ICU is implemented as a hybrid model to support the high-intensity coverage of care at a physical location, and it has been associated with improved ICU mortality, decreased length of stay, and cost-effectiveness [19-21]. Arneson et al. reported on the use of telemedicine in a clinical setting for ICU nurses to improve collaboration and minimize clinical risks to bedside nurses while maintaining a high level of care for critical patients [22]. Koubek et al. recently reported on the implementation of virtual supervision for invasive bedside procedures in the ICU during the COVID pandemic, with supervisors stationed immediately outside the unit in case of emergency while limiting exposure and PPE use [23]. They found qualitatively that such virtual supervision improved teamwork as well as comfort and autonomy of performing the procedure among the learners, despite the technological limitations (e.g., not being able to see the ultrasound image and needle aspiration at the same time). As such, while the current consensus is that virtual or tele ICU may not be a stand-alone model, it provides significant support for such needed coverage of critically ill patients, and its adaptability is expanding.

This virtual critical care elective is also the first to assess the feasibility and utility of the first-person synchronous video view for learner engagement at the bedside. First-person video-based coaching has been well established in surgical fields as a feasible modality for supplementing intraoperative learning [24]. First-person video technology has been shown to better capture details of procedures that otherwise may be limited for all but the primary surgeon or proceduralist in a direct observation setting [25]. Since the COVID-19 pandemic, many surgical specialties expanded the use of first-person video learning to augment educational opportunities for resident physicians and medical students [26]. Given the high volume of medical interventions in response to rapid changes in monitoring that occur with ventilator use, ECMO use, and hemodynamic management in an ICU setting, we incorporated the first-person view into the virtual patient interaction.

In this elective, the first-person view provided real-time critical information about the patient’s clinical presentation, physical exam, and management, all of which could not be obtained accurately and independently by the medical students with online charting only. It also provided visible, real-time interdisciplinary interaction with the bedside staff, such as nurses and respiratory therapists, which the medical students found engaging and useful for patient assessment. In addition to finding the quality of the first-person view acceptable, all nine medical students strongly agreed on the educational value of adding the first-person view to virtual patient interactions. Therefore, in the ICU setting, we believe the first-person video view to be feasible, useful, and appropriate for more widespread educational use.

The analysis of the daily ICU GS completion demonstrated both feasibility and a high level of learner engagement. Learner engagement is traditionally evaluated under three domains: behavioral (conduct, effort, and participation), affective (interest, attitude, and emotion), and cognitive (self-regulation and cognitive investment) [27]. Among these, previous virtual clerkships have assessed the behavioral and affective domains by demonstrating knowledge retention through multiple-choice tests [28-30] and post-rotation surveys of emotion and experience [30-32]. However, these assessments provide an incomplete understanding of the behavioral and cognitive domains of learner engagement.

In comparison, we found the daily ICU GS to be helpful in assessing all three domains of learner engagement. In this virtual critical care elective, the trend of the daily ICU GS completion percentages was overall sustained or increased for every student throughout the duration of the elective. Such a trend demonstrates the daily ICU GS was feasible and easily integrated into virtual rounds; otherwise, we may have seen a lower completion percentage followed by a steeper rise in completion as the medical students adjusted to the mechanics of filling out the daily ICU GS. The sustained, high level of completion suggests successful behavioral engagements as it necessitates appropriate conduct, such as active listening and comprehension, as well as significant mental effort and participation. It also demonstrates cognitive engagement; the act of filling out the multiple system-based components of the daily ICU GS alone entails a high level of cognitive investment for collecting and analyzing complex patient data as well as self-regulation to commit to the process while avoiding potential distractions that often dilute the virtual learning experience.

As for the affective domain, while the completion percentage or trend cannot directly infer interest, the medical students were instructed to utilize the daily ICU GS only if they found it helpful and were informed that its use or disuse will not affect their grades. All medical students voluntarily utilized the ICU GS and continued to do so at a high completion rate throughout the elective period, demonstrating positive and favorable interest and attitude.

Additionally, based on the survey responses and free-text qualitative feedback, most medical students found the daily ICU GS to be helpful for following along with rounds and for formulating a critical care patient assessment and plan. This suggests that the daily ICU GS added the benefit of not only measuring but also promoting learner engagement by helping early learners identify and synthesize key information. By doing so, the daily ICU GS provided a mental model for collecting clinical data to build an assessment and plan for different critical care scenarios. These results are consistent with the previously published utility of the daily ICU GS in improving provider engagement, performance, multidisciplinary communication, and patient outcomes [14,15].

Pronovost et al. demonstrated that implementing the daily ICU GS improved the understanding of short-term clinical goals among the resident physicians and nurses and also was significantly associated with decreased ICU length of stay from a mean of 2.2 days to 1.1 days. Rawat et al. demonstrated qualitative outcomes in a non-ICU inpatient setting that the use of the daily ICU GS clarified patient-centered goals, provided an accurate source of information for each patient, and improved interdisciplinary communication [15]. In all, the daily ICU GS provides a novel opportunity to assess learner engagement in a more granular way than the previous post-elective surveys have done.

After completing the course, all medical students reported via an anonymous free-text written feedback survey that they felt more prepared for in-person critical care elective and that they would recommend this elective to a colleague. Qualitative feedback revealed that the medical students felt engaged and were given ample opportunities to actively participate in patient care despite the virtual experience. They also reported learning from interactions with the interdisciplinary team in real time. The students commented that such teamwork was enhanced by the post-round debrief session through intentional reflection, sharing, and learning. The ability to create a virtual clinical learning context in which students felt that they were members of the care team and were able to meaningfully contribute to patient care was a significant accomplishment. The use of the daily ICU GS and the daily post-round debrief are examples of active learning strategies that engage students and support deeper learning and professional identity formation [33]. Based on students' feedback, the virtual critical care elective was able to improve a medical student’s engagement, preparedness, and interest in the field of critical care. 

Study limitations

There are several limitations that should be mentioned, and we hope to address them in follow-up educational interventions. The virtual critical care elective was only seven to eight days in duration for each cohort, which is much shorter than traditional in-person ICU electives. Although our study describes the largest virtual elective in an ICU setting published to date, we aim to increase the number of medical students for subsequent electives. Our virtual elective design incorporated only synchronous learning (real-time virtual rounds, post-round debrief sessions, and grand rounds) without the use of asynchronous material, such as prerecorded lectures and textbook or literature-based learning, which are also traditionally a part of an in-person elective. As we further develop the course, we plan to utilize different methods of synchronous and asynchronous learning and evaluate their independent impact on learner engagement. There is also a need to further assess the use of the daily ICU GS in both measuring and promoting learner engagement by directly comparing it to additional metrics of learner engagement, such as pre- and post-elective knowledge exams.

## Conclusions

This virtual critical care elective is the largest virtual elective reported in an ICU setting to date. It effectively utilized a first-person camera view and successfully promoted and sustained a high level of learner engagement with the use of the daily ICU GS. The medical students were able to actively care for, assess, and contribute to the clinical plan for critically ill patients as part of a multidisciplinary care team. This model may serve as the basis for the creation of virtual ICU electives elsewhere. With engaging and synchronous virtual ICU electives, educators will be able to maintain high-quality medical education in the setting of the current pandemic as well as future emergencies that may cause further inequitable access to clinical experience.

## Figures and Tables

**Figure 7:**
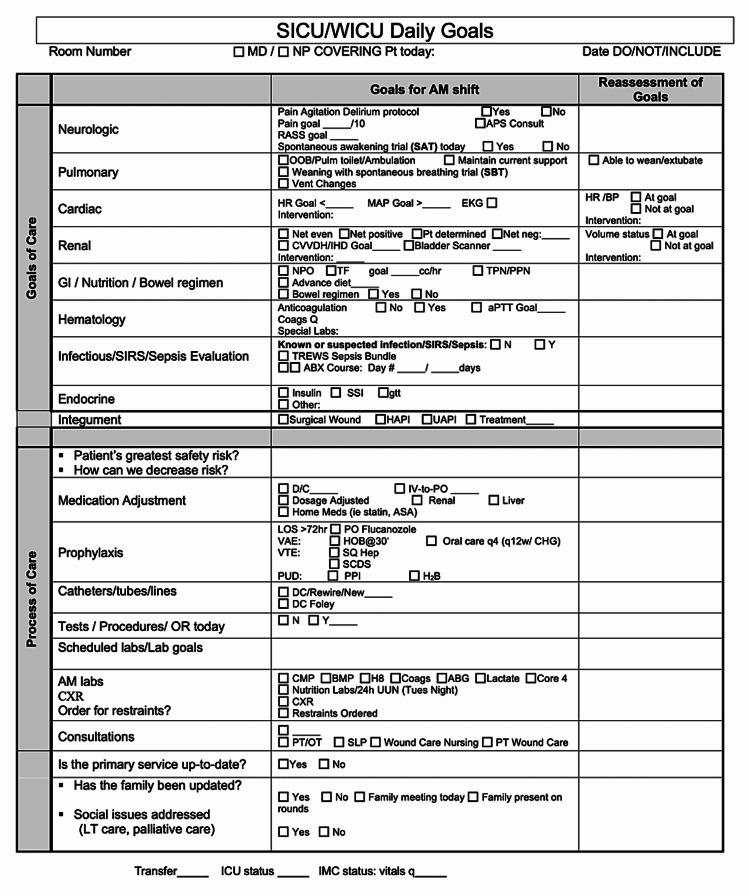
ICU daily goals sheet

**Figure 8:**
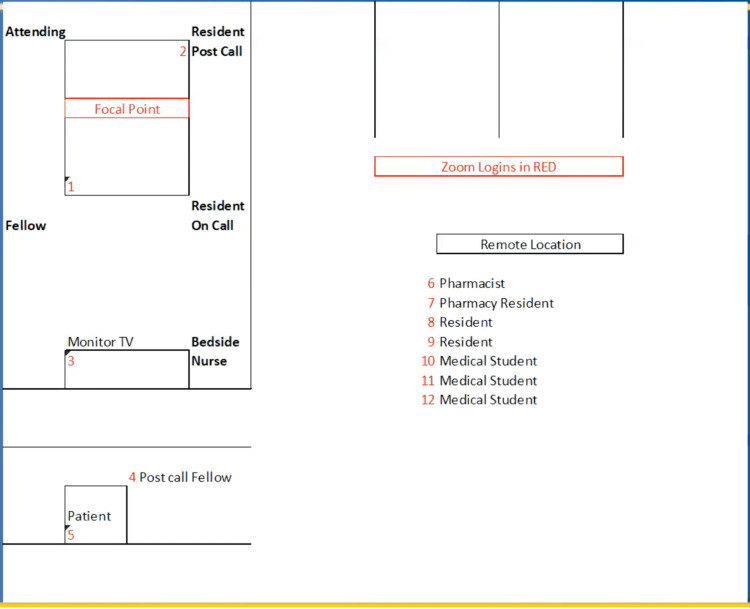
Geography of Zoom round setup

## Appendices

Data supplement: how to recreate virtual critical care elective

Please use the following links for video guidance:

1. How to mount a cell phone for Zoom connection under PAPR or over droplet precaution personal protective equipment?

a. https://vimeo.com/401415129

2. How to organize and conduct zoom critical care rounds for patient care and teaching?

a. https://vimeo.com/401406654

3. Geographical organization of Zoom ICU rounds
